# Reading Ability Development from Kindergarten to Junior Secondary: Latent Transition Analyses with Growth Mixture Modeling

**DOI:** 10.3389/fpsyg.2016.01659

**Published:** 2016-10-25

**Authors:** Yuan Liu, Hongyun Liu, Kit-tai Hau

**Affiliations:** ^1^Faculty of Psychology, Southwest UniversityChongqing, China; ^2^Key Laboratory of Cognition and Personality, Southwest University, Ministry of EducationChongqing, China; ^3^Beijing Key Laboratory of Applied Experimental Psychology, Beijing Normal UniversityBeijing, China; ^4^School of Psychology, Beijing Normal UniversityBeijing, China; ^5^Department of Psychology, Chinese University of Hong KongHong Kong, Hong Kong

**Keywords:** reading development, latent transition analysis, growth mixture model, dynamical systems, social rating

## Abstract

The present study examined the reading ability development of children in the large scale Early Childhood Longitudinal Study (Kindergarten Class of 1998-99 data; Tourangeau et al., 2009) under the dynamic systems. To depict children's growth pattern, we extended the measurement part of latent transition analysis to the growth mixture model and found that the new model fitted the data well. Results also revealed that most of the children stayed in the same ability group with few cross-level changes in their classes. After adding the environmental factors as predictors, analyses showed that children receiving higher teachers' ratings, with higher socioeconomic status, and of above average poverty status, would have higher probability to transit into the higher ability group.

## Introduction

Reading is an important activity composing of various sub-skills which grow at different speed. In reality, students are nurtured in a dynamic system where they are not only self-organizing, but also interacting and being substantially affected by the psychosocial environment (Votruba-Drzal et al., 2008; Ding et al., 2013; Iruka et al., 2014). In such a system, one under-researched area is the effect of young students' social environment at home and at school on their learning to read behavior. The purpose of the present study, therefore, was to explain the pattern of reading development and to depict the relations between the developmental pattern and children's behavior as perceived by their parents and teachers. We applied and explored with the application of the latest appropriate statistical method—the latent transition analysis with growth mixture model on a large scale longitudinal survey (Early Childhood Longitudinal Study, Kindergarten Class of 1998-99, ECLS-K, Tourangeau et al., 2009).

## Reading development: non-continuous pattern and grouping

Reading can be seen as a way of meaning extraction which requires the working of different sub-skills on the text (Stahl, 1997; Clay, 2001; Rodgers, 2004). Recent research has highlighted the need to look more closely at the different skills. Word reading, therefore, might have to be separated from reading comprehension because the former includes some of the basic phonological abilities, letter knowledge, and short-term memory (Muter et al., 2004; Kendeou et al., 2009), whereas the latter may need inference, monitoring, and knowledge of the story structure (Vellutino et al., 2007; Kendeou et al., 2009).

The mastery process of the language is, however, quite different for different subskills, such as for the constrained and unconstrained skills (e.g., Paris, 2005, 2009; Paris et al., 2005). Children's reading ability grows irregularly with spurts and stops (de Weerth et al., 1999). For example, with substantial individual differences, children's language competence may grow extremely rapidly before Spring Grade 1 but may decline thereafter (Palardy, 2010; Kieffer, 2012). Verhoeven et al. (2011) showed that the different patterns of the reading development were distinct from those around Grade 2.

Since the reading development pattern may differ from phase to phase, researchers are very interested in tracing and examining the growth trajectories. Paris (2005) suggested that when calibrating the unconstrained skills to the constrained skills, reading development follows a non-continuous growing pattern. This may not be easily detected when a simple linear growth modeling is used. Thus, for example, Quinn et al. (2015) have to use a two-part model to depict separately the developmental trajectories of the vocabulary knowledge and the reading comprehension through Grade 1 to Grade 4. Their bivariate model showed that vocabulary knowledge acted as a causal indicator of the subsequent reading comprehension growth. In summary, if researchers intend to depict the full picture of the reading developmental trajectory, a continuous growth model may not be suitable. Students stay at different “stages” with adaption to the new context using different reading skills.

A more sophisticated issue is that not all students share the same growing pattern across stages (Kaplan, 2002; Pianta et al., 2008). Empirically, these differential patterns in growth can be analyzed by (i) differentiating children into language ability groups and (ii) tracing their changes in groups as they progress in schools. For example, while most students develop rapidly before Spring Grade 1 and then slow down afterwards, some children may have a consistently slow growth rate (Kaplan, 2005; Kapland, 2008; Palardy, 2010).

The variation in growth rate is more likely to occur in the lower grades—as early as first grade (Ferrer et al., 2015), or around age of eight (Stanovich, 1986). Studies also showed that the dyslexic reader would probably grow at a slow pace that hardly enables the children to catch up with other typical readers (Grimm et al., 2010; Ferrer et al., 2015). The grouping phenomenon among slow developers is potentially harmful to them, since this low-ability-group students may have lower self-efficacy or motivation to learn let alone their ability shortage. Thus, it is important to find the conducive factors to facilitate these low ability students to “transit” into the higher competence group.

To solve the above challenging questions, we need a combined model to depict the various developing patterns with spurs and spots. Furthermore, as students' growth is determined by their current pre-exiting ability as well as by other influential factors in the environment, a dynamic systems model was adopted to analyze the interplay of these factors.

## Reading development in dynamic systems

To depict and explore the reading development, two issues should be noticed. Firstly, the sub-skills are correlated among each other. For example, Verhoeven et al. (2011) showed that the vocabulary at the beginning phase could predict word decoding and reading comprehension at the early stages of development. From Grade 2 onwards, word decoding competence in turn predicted later vocabulary development. Reading ability develops under the effects of the formal skills (Oakhill and Cain, 2012). Secondly, children live in a complicated environment where many of the external factors may influence the reading development. Thus, a *dynamic systems* view should be introduced when describing such a development.

The dynamic systems theory originates from natural science studies (for a review, see van Geert, 2003). According to this perspective, individual development is a consequence of the dynamic interactions within an individual and between an individual and the environment. In the last two decades, the dynamic systems view has been intensively discussed and widely applied, especially in language development research (Robinson and Mervis, 1999; van Geert and Steenbeek, 2005; Hollenstein, 2011; van Geert, 2011).

According to the dynamic systems, reading development can be described in terms of the *change, interactions*, and *conjoint analysis* of the individual and environment systems (Clay, 1977, 2001). For example, Clay (2001) believed that individuals would be able to construct and self-organize with their potential ability. They will push through the boundaries and improve their knowledge with their skills already mastered. So, proficient readers are able to mobilize the processing systems to fit the challenges of different texts by using environmental cues such as visual and motor stimulants. Kainz and Vernon-Feagans (2007) showed that the acquisition of reading ability was not isolated from the outside world. Kainz and Veron-Feagans worked with their colleague and developed a system of the dynamic circles involving the individuals, families, classrooms and school systems. This would be helpful to children's reading development and possibly help their transitions into higher ability groups (Kainz and Vernon-Feagans, 2007; Vernon-Feagans et al., 2008).

Among various factors in the social environment, teachers and parents' perception and attitude on students' study behaviors play important roles. These factors and their interplay vary from one individual to another and crucially affect students' academic outcomes. Ladd et al. (1999) *Child* × *Environment* model provides further explanation on how the quality of children's relationships can directly and indirectly influence school achievement from a dynamic system perspective. In the model, they show that children's initial behavior or the background factors influence their relationships with peers and teachers. Peer and teacher relationships in the school environment enhance or sometimes adversely affect student's achievement. For example, it is likely the students from lower socioeconomic backgrounds would be benefitted more by teachers who employed a more interpersonal approach of instruction, such as incorporating mixed group work, using peer tutoring, and solving problems with partners (Jung, 2014). Other studies have also consistently shown that high quality teacher-child relationship is conducive to high achievement (Davis, 2003; Pianta and Stuhlman, 2004; Hughes and Kwok, 2007; O'Connor and McCartney, 2007; Hughes et al., 2008). This relationship is also influenced by children's social behavior, such as their classroom engagement, which in turn affects children's achievement and academic outcomes (Cohen, 1997; Hughes and Kwok, 2007; O'Connor and McCartney, 2007).

From a dynamic systems perspective, teachers and parents could offer help to speed up children's transition into higher ability groups (Cho et al., 2013; Eyden et al., 2014). For example, teachers and parents' perceptions of students' ability and effort are closely related to children's academic achievement (Rytkönen et al., 2007; Natale et al., 2009; Longobardi et al., 2011). Particularly, since highly motivated children are perceived as talented and effortful (Upadyaya et al., 2012), parents and teachers' positive perceptions on children would be conducive to children's development. Upadyaya and Eccles (2015) showed that teachers' perceptions on ability and effort could predict the subsequent reading ability in a longitudinal study. It is thus quite important how teachers and parents perceive and show to the students their positive evaluation. This is because at the early elementary school years, children often assimilate teachers' perceptions in formulation the judgment of their own ability (Rosenholtz and Simpson, 1984; Tiedemann, 2000). From another perspective, children's educational aspiration partially reflected their parents and teachers' expectation on them as well, thus highlighting the importance of setting an appropriate but sufficiently high educational aspiration (Kuklinski and Weinstein, 2001; Herbert and Stipek, 2005).

## The present study

Two important issues would be addressed in the present study. Firstly, we were interested in the transition showing students' potency to develop their abilities. There are patterns shared by children in the same group in that they improved in their mastery of different reading skills, and thus grew together from one stage (lower ability groups) to the next (higher ability groups). Secondly and more importantly, we are interested in those environmental variables, especially the parents and teachers' perception on the children, that might facilitate such a transition.

Driven by the research questions, we had several research questions to examine under the dynamic systems theory. First, according to the integrated view of dynamic systems theory, a self-organizing process reflected an auto-regression development. We would examine whether and how extensive the subsequent ability status was determined by the previous status. Second, we would examine how much individual differences existed in students' growth trajectories. Finally, the contribution of parents' and teachers' perception on students' growth would be examined.

## Methods

### Participants

We used the publicly available data in the Early Childhood Longitudinal Study, Kindergarten Class of 1998-99 (ECLS-K) (Tourangeau et al., 2009)^1^ to examine our research questions. This data set was developed under the National Center for Education Statistics (NCES). We chose the ECLS-K because it focused on children's early school experiences from kindergarten to Grade 8, and the longitudinal data displayed students' long-term trajectory development. Furthermore, ECLS-K adopted a multi-source, multi-method approach, which included interviews with parents, data from principals and teachers, information from student records, and direct assessment on children (including reading, mathematics and science cognitive items). The study was in alignment with the dynamic systems theory, in which various environmental variables were considered.

**Figure 1:**
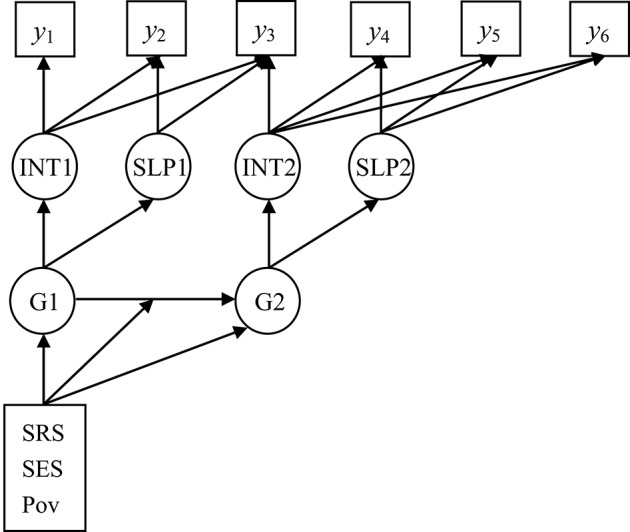
**Latent transition analysis with growth mixture model (LTA-GMM) on reading development**. The dynamic systems of reading development show the relations among the reading ability, initial state, slopes of two stages, and latent groups with social rating (SRS), SES, and poverty as the environmental factors. *y*_1_ − *y*_6_, reading ability indicators from Wave 1 to Wave 6; INT1, Stage 1 Intercept; SLP1, Stage 1 Slope; INT2, Stage 2 Intercept; SLP2, Stage 2 Slope; G1, Stage 1 Grouping (two groups); G2, Stage 2 Grouping (two groups).

In total, seven waves of measures of reading assessment were available in the data set (C1R4RSCL–C7R4RSCL). As the data at Fall Grade 1 (C3R4RSCL) contained only 30% of the total sample, without jeopardizing the generalization of our conclusion, it was not included in our study. The remaining data points were from Fall Kindergarten, Spring Kindergarten, Spring Grade 1, Spring Grade 3, Spring Grade 5 and Spring Grade 8 (y_1_−y_6_ in Figure 1). Together with the parents' and teacher's questionnaires, 7803 children's questionnaires were available in our analyses.

There were 456 individuals with missing covariate values, and totally 1033 individuals with missing values on one or more of the covariates or indicators. For the missing rate of each variable, other than the slightly higher rate at *y*_1_ (7.0%), all other ranged from 0.5 to 4.8% only, with an overall average missing rate of 2.6%. Generally, the missing pattern of the present dataset could be treated as missing at random, so that the multiple imputation method by Mplus 7.0 (Muthén and Muthén, 2012) could be appropriately used. We generated 10 datasets, and the sample size 7803 was applied to the analyses with either the null model or with covariates being included. Basic information among the variables is shown in Table 1.

**Table 1:** **Correlations and descriptive statistics of variables used in the analyses**.

	**1**	**2**	**3**	**4**	**5**	**6**	**7**	**8**	**9**	**10**
1. Parent Rating	−									
2. Teacher Rating	0.23	−								
3. SES	0.18	0.19	−							
4. Poverty	0.12	0.15	0.49	−						
5. *y*_1_	0.21	0.39	0.43	0.28	−					
6. *y*_2_	0.22	0.39	0.39	0.27	0.80	−				
7. *y*_3_	0.22	0.39	0.39	0.29	0.69	0.78	−			
8. *y*_4_	0.23	0.38	0.45	0.33	0.61	0.67	0.76	−		
9. *y*_5_	0.23	0.36	0.46	0.32	0.59	0.63	0.72	0.85	−	
10. *y*_6_	0.21	0.34	0.48	0.31	0.53	0.55	0.61	0.75	0.79	−
*M*	3.13	3.06	0.11	1.84	−1.26	−0.68	0.16	0.82	1.08	1.34
*SD*	0.22	0.42	0.63	0.14	0.26	0.24	0.19	0.09	0.08	0.15

*y_1_ − y_6_ = reading ability indicators from Wave 1 to Wave 6*.

### Measures

#### Reading ability

**Figure 2:**
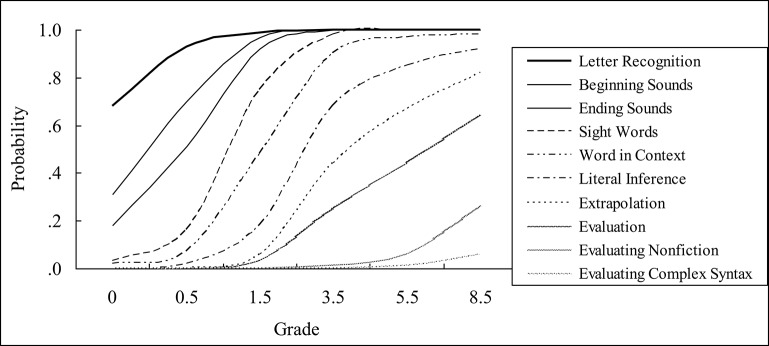
**Probability of mastery of different proficiency levels at different grades**. The ten levels of reading proficiencies were: (1) Letter Knowledge, identifying upper- and lower-case letters of the alphabet; (2) Beginning Sounds, associating letters with sounds at the beginning of words; (3) Ending Sounds, associating letters with sounds at the end of words; (4) Sight Words, recognizing common “sight” words; (5) Words in Context, reading words in context; (6) Literal Inference, making inferences using cues that were directly stated with key words in text; (7) Extrapolation, identifying clues used to make inferences; (8) Evaluation, demonstrating understanding of author's craft and making connections between a problem in the narrative and similar life problems; (9) Evaluating Nonfiction, comprehension of biographical and expository text; and (10) Evaluating Complex Syntax, evaluating complex syntax and understanding high-level vocabulary (Tourangeau et al., 2009).

The reading items were drawn from assessments used in other large-scale studies of similar-aged youth, including the National Assessment of Educational Progress (NAEP), the National Education Longitudinal Study of 1988 (NELS:88), the Education Longitudinal Study of 2002 (ELS:2002), the Texas Assessment of Knowledge and Skills (TAKS), and previous rounds of the ECLS-K. The reading items in ECLS-K were repeatedly measured with ten levels of the reading ability (see Figure 2). Each new wave was recalibrated to the former one and tests at each wave included some identical items so that the instruments at different waves could be linked on the same IRT scales (represented on the same unit of measurement). Specifically, in the collection of the Grade 8 data which was used in the present analysis, all the proficiency scores for the former levels were re-estimated to be pooled with the latest wave (see Tourangeau et al., 2009 for details).

#### Social rating

The social rating was the evaluation of the children's behavior by parents and teachers. The items were obtained from the Social Rating Scale (SRS) Approaches to Learning scales of the ECLS-K Parent and Teacher questionnaires. The SRS survey items comprised of parents' and teachers' ratings on how frequent and whether students had those study-related behaviors or not. The scale contained items such as intrinsic motivation, persistence/attention, and study habits. These ratings by teachers and parents, rather not self-reported by children, reflected students' social behaviors as perceived by the others, thus shows the interaction between students and their guardians.

A four-point scale was used, with “1 = never” and “4 = very often.” Parents' SRS was collected annually except in the third, fifth, and eighth grades, while teachers' SRS was not collected at the eighth grade. In the study, the SRS in Fall Kindergarten was used to predict the transition of latent class. All these items were used as continuous variables in the present analyses (see Tourangeau et al., 2009).

#### Background information

While many studies had investigated the relationships among Socio-economic status (SES), poverty, race, minority and achievement, which were generally used as the background variables (e.g., Hattie, 2009; OECD, 2013). Specifically, SES referred to students' relative position in the social hierarchy, directly reflected the resources at home, and was often used as an important controlling variable. Both SES and poverty status measured important characteristics of the background family information and were thus chosen in our analysis (see Tourangeau et al., 2009).

### Analyses procedure

#### Model definition

The latent transition analysis (LTA) (Prochaska and Velicer, 1997) was used to analyze the longitudinal transitions. The auto-regression part of the LTA model described appropriately the self-organizing process under the dynamic systems. LTA also allowed us to add environmental covariates to moderate the auto-regression process. With the LTA model, the measurement part could be further replaced according to different contexts and situations.

As an extension, we took advantage of the growth mixture model (GMM) to replace the measurement part of the original LTA (see Muthén et al., 2012). The GMM model could detect the growth of the reading skills by allowing individual differences in growth rate within each group, in contrast to the more stringent requirement with little individual differences allowed at each point of time.

According to earlier studies (Votruba-Drzal et al., 2008; Kieffer, 2012), the Spring Grade 1 (y_3_) was chosen to be the cut point of two stages. Thus, (y_1_−y_3_) were the indicators of Stage 1 (kindergarten stage) with latent growth factors INT 1 and SLP 1 (intercept/initial state and slope) classified into latent groups (G1); whereas (y_3_−y_6_) were the indicators of Stage 2 (primary to junior high school) with latent growth factors INT2 and SLP2 classified into groups (G2, see Figure 1).

#### Implementing the 3-step analysis

Specifically, in testing the effects due to environmental facilitating factors, covariates have to be introduced into the LTA. When adding these covariates, it is necessary to find appropriate ways to control for the characteristics that predict the membership in the different latent classes. Therefore, a *3-step Maximum Likelihood Method* (referred to the *3-step* approach in subsequent discussion) was used (see Collins and Lanza, 2010; Vermunt, 2010; Asparouhov and Muthén, 2014, see also Liu and Liu, 2015 for details).

In the first step in the 3-step LTA, GMM was used to get the classification of latent class for each stage, using the indicators at their respective stage only. For example, when estimating GMM at Stage 1, *y*_1_ to *y*_3_ were used as indicators, with *y*_4_ to *y*_6_ and the covariates serving as auxiliary variables; the proportions of each latent class were recorded. Similarly, GMM was conducted at Stage 2. In the second step, using the classification outcomes and the proportions given by Mplus, the classification error was computed for each latent class. With the odds ratio computed by the second step as the starting value of each latent class, LTA (G2 was regressed on G1) with the covariates (G1, G2, and the transition from G1 to G2, respectively, regressed on covariates) was applied (for detailed syntax, see Asparouhov and Muthén, 2014).

#### Model selection indices

The selection of the number of the latent classes has been a topic of much discussion (e.g., Nylund et al., 2007; Tofighi and Enders, 2008; Peugh and Fan, 2012). Most studies suggested that the BIC (Bayesian information criterion) value should be the best choice because it was a sample based index which also penalized sophisticated model. Tofighi and Enders (2008) in their simulation study showed that a sample size adjusted BIC (aBIC) was an even better index, and thus was used in our study. A smaller BIC/aBIC value indicated better model fit for nesting models. Besides, the entropy value was to measure how well a mixture model separated the classes. An entropy value close to 1 indicated good classification certainty. Asparouhov and Muthén (2014) suggested that an entropy level of 0.6 or higher might provide sufficient good classification for the 3-step method.

## Results

### Selection of the proper model

As LTA was used in combination with GMM, the original GMM analyses were examined first. The piecewise GMM (*y*_1_ − *y*_3_ as Piece 1 and *y*_3_ − *y*_6_ as Piece 2) null model was chosen. We conducted the exploration analyses from 2 to 4 classes (see Table 2). The model fit indices, −2*LL*, BIC, and aBIC, consistently supported a 3-class model. Then we checked the class proportion to ensure the empirical significance. For the 2-class model, the proportion was 0.95 and 0.05 for each class; for the 3-class model, the proportion was 0.93, 0.05, and 0.02; for the 4-class model, two groups contained 0 individuals. It was evident that the third group in a 3-class model was so tiny (less than 5%) and would not contribute substantially and empirically to the model, so the 2-class model was retained.

**Table 2:** **Model comparison and selection**.

	**BIC**	**aBIC**	**−2*LL***	**Entropy**
GMM_2c	17231	17170	17060	0.914
GMM_3c	16966	16893	16760	0.902
GMM_4c	17302	17216	17060	0.957
LTA-GMM_2c	11269	11171	10991	0.734
LTA-GMM_3c	12098	11958	11704	0.897
LTA-GMM_2c (3-step)	8099	8094	8082	0.915

*2c, 2 classes; 3c, 3 classes; 4c, 4 classes; 3-step, 3 step method*.

We then conducted the GMM-LTA null model, using two stages of growth but without any covariate. Results showed that the 2-class model was the best according to the selection criteria (BIC and aBIC), with slightly worse but acceptable entropy value (see Table 2).

**Figure 3:**
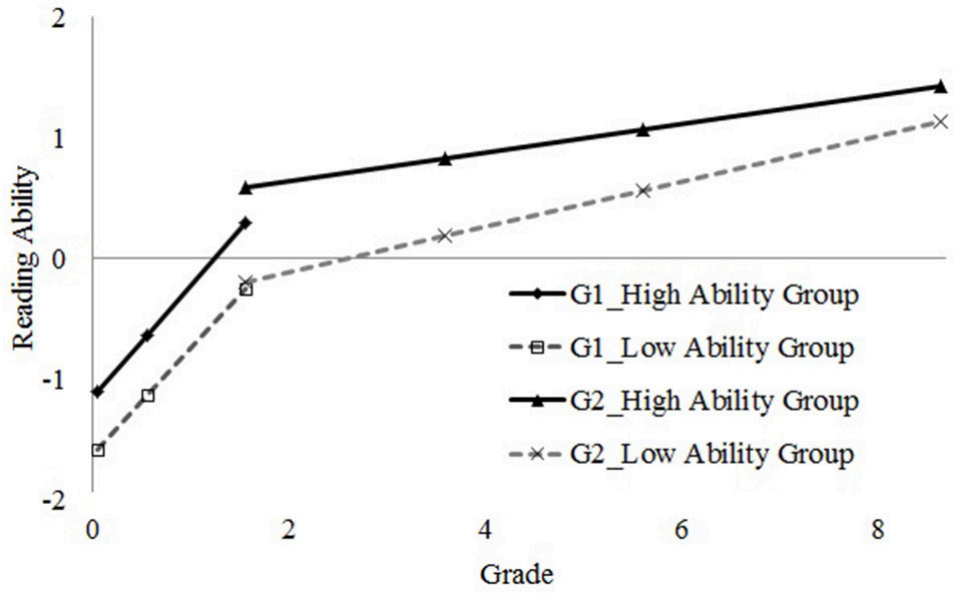
**Reading growth trajectories**. G1, Grouping at Stage 1; G2, Grouping at Stage 2.

Finally, we conducted the 3-step GMM-LTA. BIC was 8099 with an entropy value of 0.92. The information criteria and entropy value indicated that the 3-step model was the best. The final model consisted of two groups at two stages, respectively (Figure 3).

### Grouping membership

The classification results are shown in Table 3, and the parameter estimates for the growth factors are shown in Table 4. At Stage 1, most of the students were classified into the high ability group (90.9%, with initial ability of −1.10). The other 9.1% were in the low ability group with a lower initial status (−1.56). The growing rate (slope) of the high ability group (0.93) was slightly faster than that of the low ability group (0.89), but with quite similar pattern seen from the trajectory in Figure 3. For Stage 2, from Spring Grade 1 to Grade 8, children in different classes had different growing rates. There were 95.2% in the high ability group with an initial ability of 0.41 and a growth rate of 0.12, while 4.8% in the low ability group had an initial ability of −0.48 and a growth rate of 0.19.

**Table 3:** **Class counts, proportions and conditional transition probability for the final model solution**.

	**Stage 1**	**Stage 2**	**Class count**	**Class proportion**	**Transition probability**
Combination 1	High	High	7093	0.909	1.000
Combination 2	High	Low	0	0.000	0.000
Combination 3	Low	High	336	0.043	0.474
Combination 4	Low	Low	374	0.048	0.526

*High, High Ability Group; Low, Low Ability Group*.

**Table 4:** **Parameter estimates of the growth factors for the final model solution**.

	**High ability group**				**Low ability group**			
	**Est**.	***SE***	***t***	***p***	**Est**.	***SE***	***t***	***p***
**STAGE 1**								
Means								
INT 1	−1.10	0.02	−72.62	<0.001	−1.58	0.03	−59.18	<0.001
SLP 1	0.93	0.01	198.43	<0.001	0.89	0.02	60.21	<0.001
**VARIANCES**								
INT 1	0.18	0.01	34.52	<0.001	0.16	0.01	18.69	<0.001
SLP 1	0.04	0.00	11.68	<0.001	0.09	0.01	15.12	<0.001
**COVARIANCE**								
INT 1 with SLP 1	−0.05	0.00	−21.32	<0.001	−0.05	0.00	−21.32	<0.001
**STAGE 2**								
Means								
INT 2	0.41	0.00	215.30	<0.001	−0.48	0.02	−30.13	<0.001
SLP 2	0.12	0.00	463.48	<0.001	0.19	0.00	139.32	<0.001
**VARIANCES**								
INT 2	0.01	0.00	10.94	<0.001	0.01	0.00	3.93	<0.001
SLP 2	0.00	0.00	36.12	<0.001	0.00	0.00	12.13	<0.001
**COVARIANCE**								
INT 2 with SLP 2	0.01	0.00	55.28	<0.001	0.01	0.00	55.28	<0.001

After grouping, there were two groups in each stage; so four possible combinations of sub-groups were formed (Table 3). Combination 1 (90.9%), which contains individuals classified in the high ability groups at both Stages 1 and 2, had the largest proportion. Combination 4 referred to individuals classified as low ability at both stages contained 4.8% of the population. This showed that most students' growth was stable (totally 95.7% of the population). There were about 4.3% of students being classified as Combination 3, who moved from the low ability group to the high ability group across time. No individual was in Combination 2, indicating that there was no reversed pattern (changed from high ability group to low ability group).

A transition probability showed that, once classified into the high group, students would have a 100% probability staying in the high ability group thereafter. In contrast, children starting in the low group would likely be in the low ability group at Stage 2 but had a considerably high probability to transit into the high ability group at Stage 2.

### Effect of the environmental factors

We set the significant level at *p* <.001 for this study with a large sample size. Results (see Table 5) showed that the covariates could predict the Stage 1's classification. Other than the background variables, both parents and teachers' higher ratings were associated with children's higher reading ability (with the lower ability group as the reference) at Stage 1. The Stage 2's classification could be predicted positively only by the parents' rating and SES level, with higher parents' rating and SES related to better children's performance (i.e., classified in the higher ability group). In contrast, higher teachers' rating was related to lower students' performance (being classified in the lower ability group; β = −6.02, odds ratio = 0.00).

**Table 5:** **Dynamic systems model involving environmental factors**.

	**β**	***SE***	***t***	***p***	**Odds ratio**
**STAGE 1 GROUPING ON^a^**					
Parent Rating	0.65	0.06	10.82	<0.001	1.91
Teacher Rating	1.95	0.04	47.69	<0.001	7.05
SES	1.65	0.07	25.43	<0.001	5.22
Poverty	0.59	0.07	8.77	<0.001	1.81
**STAGE 2 GROUPING ON^a^**					
Parent Rating	2.58	0.16	15.80	<0.001	13.24
Teacher Rating	−6.02	1.27	−4.75	<0.001	0.00
SES	3.66	0.88	4.14	<0.001	38.79
Poverty	−8.57	3.41	−2.52	0.012	0.00
**TRANSITION (COMBINATION 3) ON^b^**					
Parent Rating	−2.77	0.18	−15.12	<0.001	0.06
Teacher Rating	5.66	1.27	4.45	<0.001	287.75
SES	−4.14	0.89	−4.67	<0.001	0.02
Poverty	8.48	3.41	2.49	0.013	4812.21

a *Classification was regressed on the covariates*.

b *Stage 2 High ability group (cf. low ability group) was regressed on the covariates in Stage 1 low ability group*.

Interactive effects with grouping transition were examined. It was found that when teachers' ratings (β = 5.66, odds ratio = 288) were more positive, then the children had a higher chance to transit from the lower to the higher ability group. Specifically, when the teachers' ratings were one unit higher, the low ability children at Stage 1 would have 288 times higher probability in transiting to the high ability group at Stage 2. However, the effects due to parents' ratings (β = −2.77, odds ratio = 0.06) and SES (β = −4.14, odds ratio = 0.02) were negligible.

## Discussion

### Developing patterns

The present study showed the advanced 3-step GMM-LTA model well described the complex longitudinal ECLS-K database set in the dynamic systems model. The developmental trend showed a fast grow from kindergarten to Spring Grade 1 and then a slowing down to a plateau on time beyond. A closer examination of the reading ability scores (Figure 2) showed that the formal five levels of reading proficiency were more related to Paris's constrained skills which were close perfection after Spring Grade 1. After this time spot, students continuously learned unconstrained skills. From Table 4, statistical evidence showed that the variances were much smaller at Stage 2 than those at Stage 1, especially for their growth rates which had little variance at Stage 2. This indicates the non-normal distribution across the development from kindergarten to Grade 8. It is necessary, therefore, to analyze the reading skills separately at different stage, where sub-skills developed with quite different speeds and patterns.

The grouping results were consistent with the literature (Grimm et al., 2010; Ferrer et al., 2015) in that two groups with different ability levels could be differentiated. The classification indicates that most of the students were classified in high ability group, either at Stage 1 or Stage 2. We can thus treat the high ability group as the reference “normal” developing pattern, since it contained more than 90% of the population. So, students classified in the lower ability group were those likely to have reading problems. According to Ferrer et al. (2015), the grouping differentiation could emerge as early as Grade 1; our study indicates that the grouping may emerge even earlier. However, students still had a considerable chance to transit into the higher ability group through the self-organizing progress (conditional probability was 0.47). Educators should pay more attention to children's early reading problems as early as possible before they develop into more serious language learning problems.

### Environmental facilitators

We found that all the factors being examined had substantial effects on the grouping at Stage 1. Contradictory results were found, however, in the prediction of Stage 2 grouping/transition. The results showed that, parents' rating and SES positively predicted Stage 2 grouping, whereas they negatively predicted the transition. Vice versa, teachers' rating negatively predicted classification, but positively predicted the transition. These contradictions may reflect problems in the long-term prediction efficiency. When we took the transition prediction terms out of our model, all predictions on Stage 2 grouping showed negative estimates (ranged from −0.48 to −0.10), but with quite small or non-significant effects (odds ratios ranged from 0.70 to 0.91). So the social environmental variables collected at Wave 1 may have less predictive power to the subsequent ability, especially for a long-term growth (8.5 years). This is somehow similar to the previous study (Upadyaya and Eccles, 2015) which showed that teachers' perception of the effort of students could predict the subsequent reading ability with a small interval (1 year) only. Further investigations on the prediction power in long term studies would be useful.

As for the transition, the results showed that teachers' ratings had larger effects in predicting the transition probability than that of the parents'. This reveals that the teachers' ratings are probably more accurate as compared to those of the parents', which might be explained by the Child × Environment model (Ladd et al., 1999; Pianta and Stuhlman, 2004). To illustrate, the teacher-student relationship is a mediator influenced by the effect of school behavior and other background or cognitive variables on children's achievement. With the accurate perceptions, teachers may adopt more efficient approaches on students' learning. Teachers' interaction with students is thus playing as a *proximal factor* influencing the achievement influencing academic achievement more directly, while school entries (family variables) are *distal factors*. On the other hand, longitudinal studies show that teachers' perception of the students (either ability or effort) can predict subsequent children's self-concept (Natale et al., 2009); teachers are significant socialization agents whose perception greatly impact children's self-concept formation (Madon et al., 2001), and thus have a great impact on students ability. To summarize, we are alerted again of the important role of the teacher-student relationships, since students spend more time in school with their teachers when they progress in schools. In contrast, their after-class activities with parents may reduce so that the parents' evaluations become less accurate and predictive of children's reading performance.

As for background variables, SES is a potentially useful predictor of children's reading performance, particularly on grouping but not on transition. Meta-analysis (e.g., Hattie, 2009) showed that SES has a moderate impact (*d* = 0.57) on academic achievement. In the present research, we took SES as one of the important home background variables, used it as a controlling covariate, and showed that it had influence on grouping. It is logical, therefore, to pay greater attention to the reading development of students from lower SES background (e.g., Ladd et al., 1999; Jung, 2014).

## Limitations and future directions

One possible limitation is that we used a two-stage model to analyze the data. This was mainly decided from the general trajectory of the reading growth of the data and findings from earlier studies (Kaplan, 2005; Kapland, 2008; Palardy, 2010; Kieffer, 2012). However, the problem is that the interval of the stage (especially at Stage 2) is quite large with the time points of data collection being several years apart. There is a possibility, therefore, that students grow in discernible stages crossing a long period of time. If the intervals of the data collection had been much smaller, we would have been more confident to use the growth modeling within each stage. An alternative is to use the non-linear model to build the GMM (e.g. Grimm et al., 2010). But it requires demanding measures. Future studies could further explore the possible trajectories of reading development, identify the proper cutoff for each stage, and describe the most suitable trend within each stage.

We also notice that long-term effects and growth patterns are less well predicted by the social environmental covariates. These covariates may include the home and teachers' social environmental factors which generally have smaller effects than those of more direct variables such as teaching and school (for meta-analysis, see Hattie, 2009). One possible direction of the future study is, therefore, to focus on the short-term prediction of a set of more comprehensive social environmental factors from schools (teachers, peers, etc.) and families (parents, etc.). Another possibility is to treat the covariate as a time-varying variable in multilevel structure (Vermunt et al., 1999; Bartolucci et al., 2011). That means, in our analyses, the social rating recorded at Kindergarten, Spring Grade 1 Spring and Fall Grade 5 can all be treated as multiple indicators affecting the transition at different time points. Especially under the condition with a large interval of measuring time, time-varying measures would then produce more accurate prediction.

## Conclusions

In summary, the study contributes in showing that: (i) the LTA-GMM fitted the data well; (ii) most of the children stayed in the same ability group with practically few cross-level class changes in the transition; (iii) children receiving higher teachers' ratings and with higher SES, and of above average poverty status, would have higher chance to transit into the higher ability level group. The findings supported the importance of the moderating effects of these social environmental facilitators on the patterns of children's reading development.

## Author contributions

YL contributes the most to the article. HL is the corresponding author who organizes and helps conducting the analysis. KH helps a lot providing useful suggestions on modeling and revising the article.

## Funding

The manuscript is supported by the National Natural Science Foundation of China (No. 31571152).

### Conflict of interest statement

The authors declare that the research was conducted in the absence of any commercial or financial relationships that could be construed as a potential conflict of interest.

## Footnotes

^1^Retrieved from http://nces.ed.gov/ecls/kindergarten.asp.
